# Effect of sequential embryo transfer on *in vitro* fertilization and embryo transfer outcomes: a systematic review and meta-analysis

**DOI:** 10.3389/fmed.2023.1303493

**Published:** 2023-12-19

**Authors:** Wending Teng, Hong Xian, Fang Wang, Yicheng Wang, Xiangqian Meng, Xiaojian Zhang, Xudong Shan, Jiangying Yi

**Affiliations:** ^1^Department of Reproductive Health and Infertility, Chengdu Women’s and Children’s Central Hospital, School of Medicine, University of Electronic Science and Technology of China, Chengdu, China; ^2^Chengdu Xi’nan Gynecological Hospital, Chengdu, China; ^3^Sichuan Academy of Medical Sciences of Sichuan Provincial People’s Hospital, Chengdu, China; ^4^Chengdu Third People’s Hospital, Chengdu, China

**Keywords:** sequential embryo transfer, *in vitro* fertilization-embryo transfer, repeated implantation failure, systematic review, IVF

## Abstract

**Background:**

Sequential embryo transfer has been recognized as a strategy to increase pregnancy rates in women undergoing *in vitro* fertilization and embryo transfer (IVF-ET). However, its impact on assisted reproductive outcomes remains to be substantiated by robust evidence. This systematic review aims to summarize and analyze the available evidence to investigate the effect of sequential embryo transfer on assisted reproductive outcomes.

**Methods:**

A comprehensive literature search was executed across the Pubmed, Cochrane Library, Web of Science, and Scopus databases in accordance with the Preferred Reporting Items for Systematic Reviews and Meta-Analyses (PRISMA) guidelines. Data were aggregated utilizing a random effects model, and the resultant outcomes were articulated as odds ratios (ORs) along with their 95% confidence intervals (CIs).

**Results:**

The pooled results revealed a statistically significant enhancement in reproductive outcomes for infertile patients undergoing sequential embryo transfer as evidenced by elevated rates of chemical pregnancy (OR = 1.67, 95% CI = 1.23–2.27), clinical pregnancy (OR = 1.78, 95% CI = 1.43–2.21), and ongoing pregnancy (OR = 1.54, 95% CI = 1.03–2.31). Compared with cleavage-stage embryo transfer, sequential transfer yielded superior outcomes in terms of chemical pregnancy rate (OR = 2.08, 95% CI = 1.35–3.19) and clinical pregnancy rate (OR = 1.78, 95% CI = 1.37–2.31). Furthermore, among the repeated implantation failure (RIF) cohort, sequential embryo transfer surpassed blastocyst-stage transfer, delivering a heightened chemical pregnancy rate (OR = 1.66, 95% CI = 1.19–2.53) and clinical pregnancy rate (OR = 1.65, 95% CI = 1.19–2.27).

**Conclusion:**

Our meta-analysis indicates that sequential transfer may enhance clinical pregnancy rate in a small subgroup of well-selected women. While promising, further evidence from prospective studies is needed.

## Introduction

*In vitro* fertilization and embryo transfer (IVF-ET) is an important treatment option for infertile couples. However, implantation rates of 25–40% mean that many infertile couples require multiple cycles of treatment to achieve a successful pregnancy (1). This makes IVF-ET a time-consuming and costly assisted reproductive technology.

Embryo implantation is a multifaceted process. Success hinges on three pivotal elements: high-quality embryos, a receptive endometrium, and optimal synchronization between the embryo and endometrial lining (2). Among these factors, the endometrium is often deemed the most crucial, accounting for approximately two-thirds of implantation failures (3). Sequential embryo transfer, first introduced by Abramovici et al., (4) comprises two stages of embryo transfer on separate days within the same treatment cycle—specifically, the first transfer occurs on the second day post-oocyte retrieval, and the second on the third-day (4). This approach capitalizes on the “implantation window,” enhancing endometrial receptivity (4). Building on this innovative strategy, Goto et al. refined the technique by initiating the first transfer with a cleavage-stage embryo, followed by a blastocyst for the second transfer (5). This modified protocol mitigates the risk of cycle cancelation due to failed blastocyst formation and yields superior implantation and pregnancy rates compared to conventional transfer methods (5).

Repeated implantation failure (RIF) is typically defined as the inability to achieve a clinical pregnancy after transferring at least four high-quality embryos across a minimum of three fresh or frozen–thawed embryo transfer cycles in women under the age of 40 (6). One of the most critical factors contributing to RIF is likely poor endometrial receptivity (7). As such, many specialists posit that sequential embryo transfer could be an important therapeutic strategy for patients experiencing RIF.

The effectiveness of sequential embryo transfer remains a subject of debate due to insufficient published data. This study aimed to systematically review and synthesize the existing clinical evidence to elucidate the impact of sequential embryo transfer on IVF-ET outcomes, intending to refine embryo transfer strategies.

## Materials and methods

This systematic review and meta-analysis was conducted in accordance with the Preferred Reporting Items for Systematic Reviews and Meta-Analyses (PRISMA) guidelines.

### Literature search and data extraction

A comprehensive literature search was conducted using four established databases: PubMed, Cochrane Library, Web of Science, and Scopus, covering articles from their inception to July 31, 2023. We also manually screened the reference lists of selected articles to identify additional pertinent studies. The primary search terms employed were [“sequential embryo transfer” OR “consecutive embryo transfer” OR “interval double transfer” OR “two-step transfer”] AND [“*in vitro* fertilization” OR “IVF” OR “assisted reproductive techniques” OR “ART” OR “intracytoplasmic sperm injections” OR “ICSI”]. The search was not limited by language, geographical location, or study type, thereby maximizing the inclusivity and generalizability of our findings.

After conducting the literature search, two authors (WT and HX) independently screened the titles, abstracts, and full-text articles to identify eligible studies. Any disagreements arising during this process were resolved through consultation with a third author (FW), ensuring a consensus was reached. Additionally, the quality of the included studies and the overall quality of evidence were independently evaluated by two authors (WT and HX). Should disagreements occur regarding the type or quality of a given study, a consensus was reached through discussion with the third author (FW).

### Eligibility criteria

Studies had to meet three predetermined criteria to be included in this review. Firstly, the study design had to be either a retrospective cohort study, a *post hoc* analysis, or a randomized controlled trial (RCT), as these study types best align with the objectives of this review. Secondly, the studies were required to compare the outcomes of sequential embryo transfers involving both cleavage-stage and blastocyst-stage embryos against other transfer protocols. Lastly, the clinical pregnancy rates had to be reported as an outcome measure. Exclusion criteria encompassed other study designs such as reviews, case reports, and studies lacking data on clinical pregnancy rates.

### Data extraction

For each selected study, we recorded the following variables: the first author, year of publication, country of origin, study design, and patient characteristics—such as age, body mass index (BMI), embryo transfer protocol, and RIF status. Additionally, we documented any outcome metrics reported by the study.

### Outcome measures

The primary outcome measure selected for this review was the pregnancy rate, subdivided into chemical pregnancy rate, clinical pregnancy rate, and ongoing pregnancy rate. Other outcomes encompassed implantation rate, multiple pregnancy rate, and miscarriage rate.

### Assessment of heterogeneity

We assessed the similarity between the eligible studies in their design and clinical characteristics using the *I*^2^ statistic. An *I*^2^ > 50% was labeled as marked heterogeneity (8).

### Quality and risk of bias assessment

The integrity of the included studies was rigorously evaluated based on the following parameters: random sequence generation, allocation concealment, blinding of participants and personnel, blinding of outcome assessment, treatment of incomplete outcome data, and selective reporting. We employed the Grading of Recommendations Assessment, Development, and Evaluation (GRADE) approach to grade the quality of evidence systematically (9). The evidence quality was downgraded by one level of severe reservations and by two levels for exceedingly grave concerns concerning the risk of bias, inconsistency, indirectness, imprecision, and publication bias. Two authors independently executed this assessment of bias and quality. Any divergences in opinion were resolved either through discussion or consultation with a third author. The findings of this quality assessment are delineated in Table S1.

### Data analysis

Studies that satisfied the inclusion criteria were included in the meta-analysis. Data extracted from these studies were aggregated using Review Manager software (Version 5.4; The Cochrane Collaboration, 2020). Pooled odds ratios (OR) with 95% confidence intervals (CI) were calculated employing a random-effects model. Heterogeneity across studies was quantified using the I^2^ statistic. Subgroup analyses were conducted based on the RIF status of patients and whether the control group involved blastocyst-stage transfers. Additionally, sensitivity analyses were carried out by sequentially omitting studies with high weight to assess their influence on heterogeneity and effect estimates. Finally, the potential for publication bias was evaluated using funnel plots.

## Results

### Results of the searches

**Figure 1:**
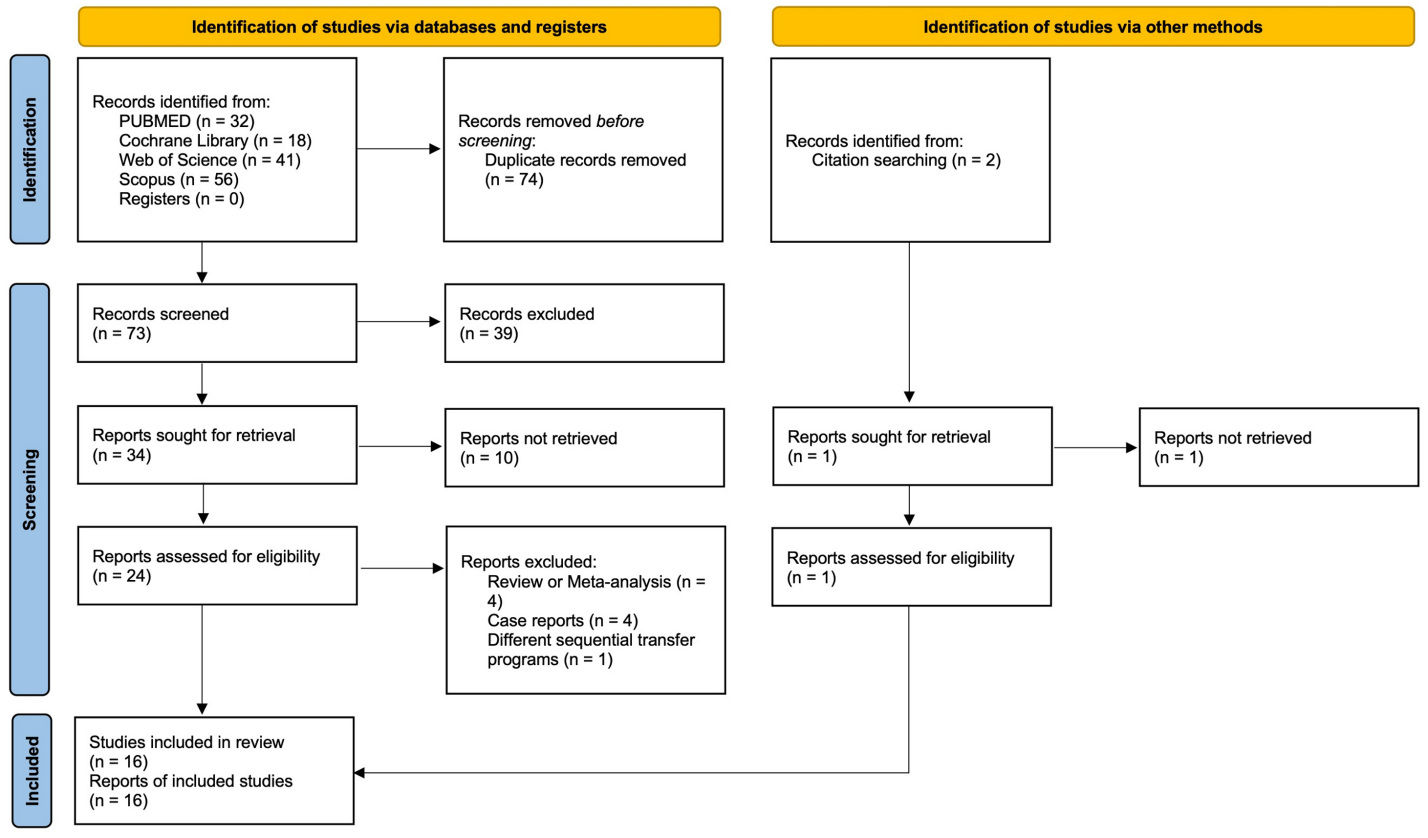
PRISMA flow diagram of the systematic search.

Our initial database search yielded 147 studies, of which 74 were duplicates and subsequently removed. After screening titles and abstracts, 34 studies were deemed eligible for further evaluation. Of these, eight were excluded due to the unavailability of full text or missing information. Detailed examination of the remaining 26 full-text studies led to the inclusion of 15 articles in the meta-analysis. An additional study that met the inclusion criteria was identified through cross-referencing, bringing the final count to 16 studies incorporated into this meta-analysis (5, 10–24). The process for study selection is detailed in the PRISMA flowchart provided in Figure 1.

### Included studies

The general characteristics of the 16 studies are shown in Table 1. Four of these were randomized controlled trials (RCTs), while the remaining 12 were case–control trials (CCTs). The studies were geographically diverse, featuring participants from North America, Africa, Asia, and Europe from 1997 to 2021. The cumulative sample consisted of 4,054 participants: 1,185 in the sequential transfer group and 2,869 in the control group. In addition, Table 1 also includes information on the patient’s age, BMI, fertilization method, cycle type, and RIF status. Notably, not all studies reported all the predefined outcomes; the outcomes reported per study are further elaborated in Table 2.

**Table 1:** Characteristics of included studies.

Study	Country	Study period	Study design	Study groups (n)	Patients’ Age (y, Mean ± SD)	Patients’ BMI (Mean ± SD)	Fertilization method	Cycle Type	RIF	Note
Antonio Sanoja Breña. (2016)	Spanish	2012.09–2014.09	CCT (RC)	D3/D5-6 sequential transfer (58) D3 single transfer (118) D5-6 single transfer (26) D3 double transfer (236) D5-6 double transfer (33) D3 triple transfer (316)	D3/D5-6 sequential transfer (36.34 ± 4.78) The age of the remaining five groups of patients is unknown.	N/A	IVF/ICSI	Fresh-ET	No	
B Almog et al. (2008)	Israel	2003.03–2005.05	CCT (RC)	D2-3/D5 consecutive transfer (65) D2-3 embryo transfer (66)	D2-3/D5 consecutive transfer (34.3 ± 0.7) D2-3 embryo transfer (34.7 ± 0.1)	N/A	IVF/ICSI	Fresh-ET	Yes	The number of embryos transferred per patient is uncertain.
Chadi Yazbeck et al. (2013)	France	2005.01–2006.12	CCT (RC)	D2-3/D5-6 consecutive transfer (120) D2-3 embryo transfer (280)	D2-3/D5-6 consecutive transfer (33.07 ± 4.13) The age of the patients in D2-3 embryo transfer group is uncertain.	N/A	IVF/ICSI	Fresh-ET	No	The number of embryos transferred per patient is uncertain.
Cong Fang et al. (2013)	China	2010.08–2011.12	CCT (RC)	D2/D3 sequential transfer (33) D3/D5 sequential transfer (57) D3 embryo transfer (85) D5 blastocyst transfer (24)	D2/D3 sequential transfer (35.1 ± 4.1) D3/D5 sequential transfer (34.1 ± 3.2) D3 embryo transfer (33.9 ± 4.1) D5 embryo blastocyst (33.1 ± 4.5)	N/A	IVF/ICSI	Fresh-ET	Yes	Exclude data from the D2/D3 sequential transfer group. The number of embryos transferred per patient is uncertain.
Ensieh Shahrokh Tehraninejad et al. (2019)	Iran	2016.04–2017-04	RCT (PC)	D3/D5 sequential transfer (60) D5 blastocyst transfer (60)	D3/D5 sequential transfer (35.03 ± 4.35) D5 blastocyst transfer (34.09 ± 4.20)	N/A	ICSI	Fresh-ET	Yes	Three embryos were transferred to patients in D3/D5 sequential transfer group, and two embryo was transferred to patients in D5 embryo transfer group.
J Ashkenazi et al. (2000)	Israel	1997.10–1998.12	CCT (RC)	D2/D3 sequential transfer (107) D2-3/D5-6 sequential transfer (29) D2-3 embryo transfer (139)	D2/D3 sequential transfer (31.1 ± 4.9) D2-3/D5-6 sequential transfer (29.5 ± 2.9) D2-3 embryo transfer (32.2 ± 5.6)	N/A	IVF/ICSI	Fresh-ET	No	Exclude data from the D2/D3 sequential transfer group. Three embryos were transferred to patients in D2-3/D5-6 sequential transfer group and D2-3 embryo transfer group.
Kaya Gözde et al. (2020)	Turkey	2011.01–2014.01	CCT (RC)	D2/D3 consecutive transfer (54) D3/D5 consecutive transfer (53) D2-3 double transfer (135)	D2/D3 consecutive transfer (32.0(28.7–35.0)) D3/D5 consecutive transfer (29.0 (26.0–33.7)) D2-3 double transfer (31.0 (28.0–35.0))	D2/D3 consecutive transfer (25.2 ± 5.2) D3/D5 consecutive transfer (24.6 ± 4.0) D2-3 double transfer (24.4 ± 4.2)	ICSI	Fresh-ET	No	Exclude data from the D2/D3 sequential transfer group. All patients included in the study were transferred two embryos.
Koichi Kyono et al. (2003)	Japan	2001.01–2002.12	CCT (RC)	D3/D5 sequential transfer (125) D3 embryo transfer (523) D5 blastocyst transfer (87)	D3/D5 sequential transfer (35.4 ± 4.5) D3 embryo transfer (35.5 ± 4.8) D5 blastocyst transfer (35.5 ± 4.5)	N/A	IVF/ICSI	Fresh-ET	Yes	The number of embryos transferred per patient is uncertain.
Mengxia Ji et al. (2022)	China	2017.01–2021.07	CCT (RC)	D3/D5 sequential transfer (77) D3 embryo transfer (154) D5 blastocyst transfer (80)	D3/D5 sequential transfer (33.39 ± 3.89) D3 embryo transfer (33.63 ± 3.97) D5 blastocyst transfer (32.50 ± 3.79)	D3/D5 sequential transfer (21.38 ± 2.72) D3 embryo transfer (21.53 ± 2.61) D5 blastocyst transfer (21.16 ± 2.65)	N/A	FET	Yes	All patients included in the study were transferred two embryos.
Ronit Machtinger et al. (2006)	Israel	1999.01–2004.05	CCT (RC)	D3/D5-6 sequential transfer (66) D3 embryo transfer (117)	D3/D5-6 sequential transfer (30.7 ± 3.2) D3 embryo transfer (31.0 ± 2.9)	N/A	IVF/ICSI	Fresh-ET/ FET	Yes	The number of embryos transferred per patient is uncertain.
S al-Hasani et al. (1990)	Germany	N/A	CCT (RC)	D2/D5 sequential transfer (38) D2 embryo transfer (68)	N/A	N/A	N/A	Fresh-ET	No	Four or five embryos were transferred to patients in D2/D5 sequential transfer group, and three embryo was transferred to patients in D2 embryo transfer group.
Saghar Salehpour et al. (2023)	Iran	N/A	RCT (PC)	D3/D5 sequential embryo transfer (102) D5 double blastocyst transfer (100)	D3/D5 sequential embryo transfer (33.92 ± 0.4794) D5 double blastocyst transfer (34.90 ± 0.5192)	D3/D5 sequential embryo transfer (26.93 ± 0.2366) D5 double blastocyst transfer (26.47 ± 0.2350)	ICSI	FET	Yes	All patients included in the study were transferred two embryos.
Sakae Goto et al. (2005)	Japan	2000.04–2003.12	CCT (RC)	D2/D5 consecutive transfer (51) D2 embryo transfer (90)	D2 embryo transfer (37.7 ± 4.8) The age of the patients in D2/D5 consecutive transfer group is uncertain.	N/A	IVF/ICSI	Fresh-ET	No	Patients in the consecutive transfer group who did not undergo a second transfer because no blastocysts were obtained were excluded. All patients included in the study were transferred two embryos.
Simon J Phillips et al. (2003)	Canada	2001.01–2002.07	CCT (RC)	D3/D5 consecutive transfer (110) D3 single transfer (32)	consecutive transfer (34.7 ± 3.48) D3 single transfer (35 ± 4.36)	N/A	IVF/ICSI	Fresh-ET	No	Two embryos were transferred to patients in D3/D5 consecutive transfer group, and one embryo was transferred to patients in D3 single transfer group.
Soheila Arefi et al. (2022)	Iran	2020.01–2021.09	RCT (PC)	D3/D5 sequential transfer (100) D5 blastocyst transfer (100)	D3/D5 sequential transfer (35.06 ± 4.33) D5 blastocyst transfer (33.90 ± 4.00)	D3/D5 sequential transfer (25.95 ± 3.75) D5 blastocyst transfer (25.83 ± 3.01)	ICSI	FET	Yes	Two or three embryos were transferred to patients in D3/D5 sequential transfer group, and two embryo was transferred to patients in D5 embryo transfer group.
Wael A. Ismail Madkour et al. (2015)	Egypt	2008.04–2011.03	RCT (PC)	D3/D5 sequential transfer (74) D3 embryo transfer (73)	D3/D5 sequential transfer (34.4 ± 1.4) D3 embryo transfer (34.0 ± 1.5)	D3/D5 sequential transfer (23.47 ± 7.92) D3 embryo transfer (21.98 ± 5.86)	ICSI	Fresh-ET	Yes	All patients included in the study were transferred three embryos.

CCT, case–control trial; ET, embryo transfer; FET, frozen embryo transfer; ICSI, intracytoplasmic sperm injection; IVF, in vitro fertilization; PC, prospective cohort; RC, retrospective cohort; RCT, randomized controlled trial; RIF, repeated implantation failure.

**Table 2:** Outcomes reported per included study.

Study	Chemical pregnancy rate	Implantation rate	Multiple pregnancy rate	Ectopic pregnancy rate
Antonio Sanoja Breña. (2016)	No	Yes	Yes	No
B. Almog et al. (2008)	Yes	No	Yes	No
Chadi Yazbeck et al. (2013)	No	Yes	Yes	No
Cong Fang et al. (2013)	No	Yes	Yes	No
Ensieh Shahrokh Tehraninejad et al. (2019)	Yes	No	Yes	No
J. Ashkenazi et al. (2000)	No	Yes	No	No
Kaya Gözde et al. (2020)	No	No	No	No
Koichi Kyono et al. (2003)	No	Yes	Yes	No
Mengxia Ji et al. (2022)	No	Yes	Yes	No
Ronit Machtinger et al. (2006)	No	Yes	Yes	No
S al-Hasani et al. (1990)	No	Yes	Yes	No
Saghar Salehpour et al. (2023)	Yes	Yes	Yes	Yes
Sakae Goto et al. (2005)	No	Yes	Yes	No
Simon J. Phillips et al. (2003)	Yes	Yes	Yes	Yes
Soheila Arefi et al. (2022)	No	Yes	Yes	No
Wael A. Ismail Madkour et al. (2015)	Yes	Yes	Yes	No

### Outcomes

#### Main outcomes

**Figure 2:**
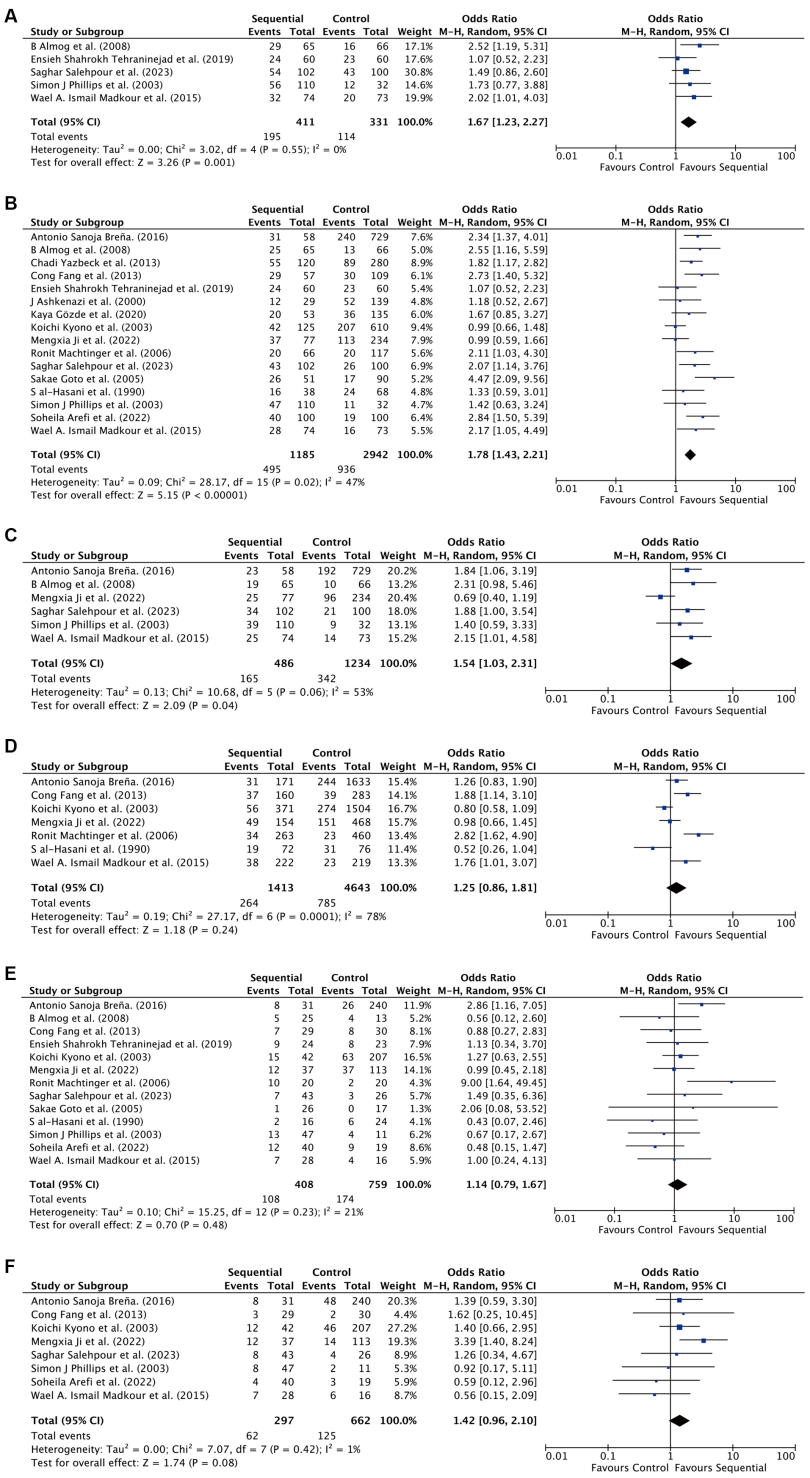
Differences in assisted reproductive outcomes between sequential transfer group and control group. **(A)** Chemical pregnancy rate. **(B)** Clinical pregnancy rate. **(C)** Ongoing pregnancy rate. **(D)** Implantation rate. **(E)** Multiple pregnancy rate. **(F)** Miscarriage rate.

**Figure 3:**
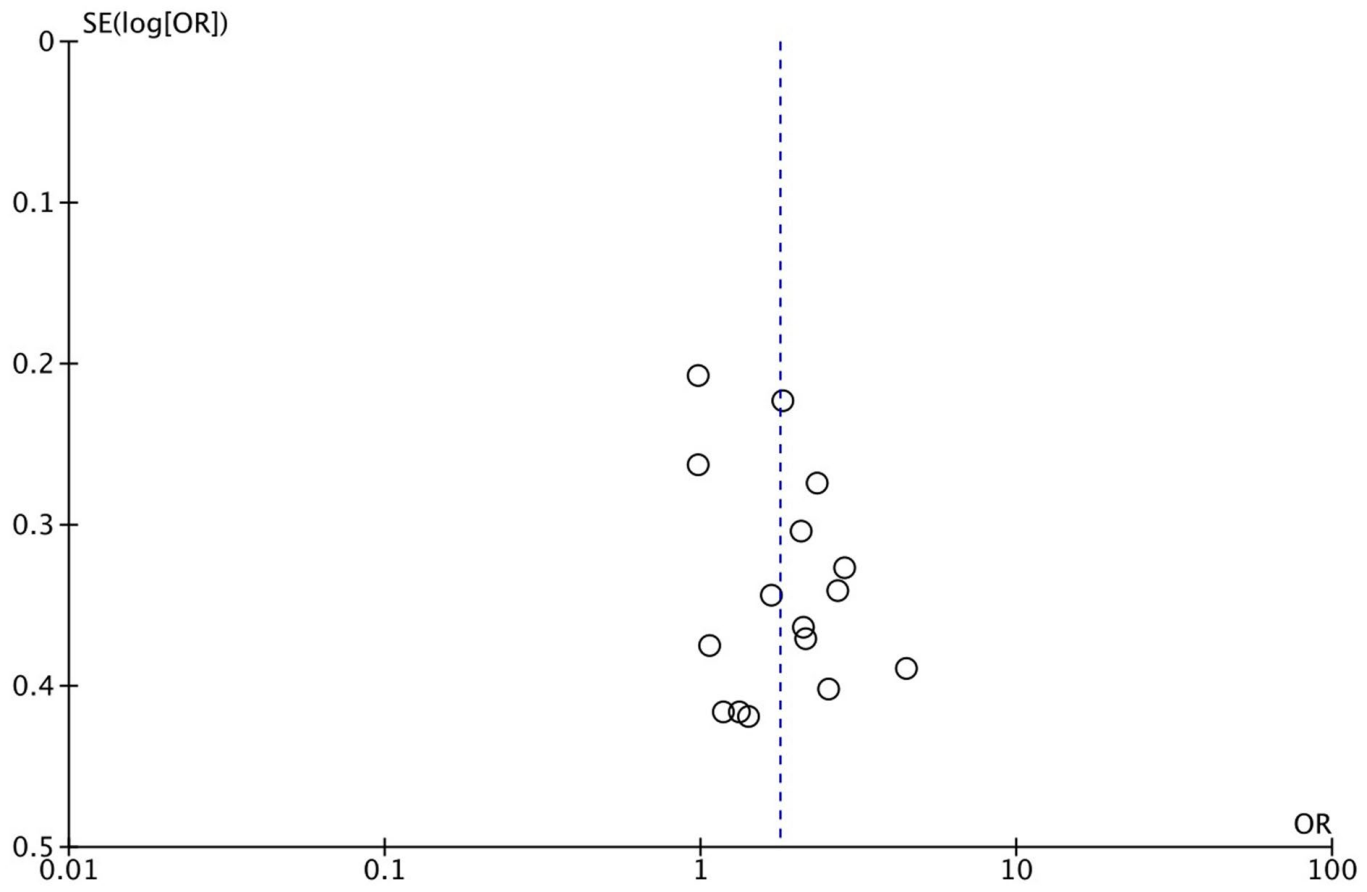
Funnel plot for assessing publication bias in systematic reviews.

For all patients included in the study, significant improvements were observed in the sequential transfer group compared to the control group for the following outcome measures: chemical pregnancy rate (OR 1.67; 95% CI 1.23–2.27), clinical pregnancy rate (OR 1.78; 95% CI 1.43–2.21), and ongoing pregnancy rate (OR 1.54; 95% CI 1.03–2.31). Conversely, no significant differences were discerned in implantation rate (OR 1.25; 95% CI 0.86–1.81), multiple pregnancy rate (OR 1.14; 95% CI 0.79–1.67), or miscarriage rate (OR 1.42; 95% CI 0.96–2.10) between the two groups (Figure 2). Analysis revealed no significant publication bias among the studies included (Figure 3). Sensitivity analyses were conducted for variables with I^2^ > 50%, and pooled adjusted ORs yielded estimates congruent with the primary findings (Supplementary Figure S1). Given the high study quality of the RCTs, we performed a separate small subgroup analysis of the four RCTs included in the study, with results similar to the overall analysis (Supplementary Figure S2).

#### Subgroup analysis

When patients undergoing only cleavage embryo transfers served as the control group, we observed a statistically significant elevation in both the chemical pregnancy rate (OR 2.08; 95% CI 1.35–3.19) and the clinical pregnancy rate (OR 1.78; 95% CI 1.37–2.31) within the sequential transfer group. However, no significant differences were found in ongoing pregnancy rate (OR 1.48; 95% CI 0.94–2.34), implantation rate (OR 1.32; 95% CI 0.86–2.03), multiple pregnancy rate (OR 1.38; 95% CI 0.87–2.18), or miscarriage rate (OR 1.49; 95% CI 0.93–2.37) between the two groups (Table 3 and Supplementary Figure S3). Sensitivity analysis corroborated these findings (Table 3).

**Table 3:** Summary of meta-analyses of comparison between sequential transfer and cleavage embryo transfer.

Outcome indicator	Samples	OR (95% Cl)	*p* value	Heterogeneity	Adjusted OR (95% Cl)	Adjusted *p* value
Chemical pregnancy rate	420	2.08 (1.35–3.19)	0.0008	0%	2.12 (1.22–3.66)	0.007
Clinical pregnancy rate	3,355	1.78 (1.37–2.31)	< 0.0001	53%	1.91 (1.50–2.43)	< 0.0001
Ongoing pregnancy rate	1,379	1.48 (0.94–2.34)	0.09	53%	1.43 (0.78–2.60)	0.25
Implantation rate	5,528	1.32 (0.86–2.03)	0.20	82%	1.47 (0.93–2.33)	0.10
Multiple pregnancy rate	904	1.38 (0.87–2.18)	0.18	25%	1.40 (0.79–2.47)	0.25
Miscarriage rate	743	1.49 (0.93–2.37)	0.09	9%	1.40 (0.73–2.68)	0.31

When utilizing patients who underwent only blastocyst transfers as the control, the clinical pregnancy rate (OR 1.68; 95% CI 1.12–2.53) was the sole metric to display a significant difference between the groups. Neither the chemical pregnancy rate (OR 1.32; 95% CI 0.85–2.06), ongoing pregnancy rate (OR 1.59; 95% CI 0.61–4.13), implantation rate (OR 0.98; 95% CI 0.67–1.41), multiple pregnancy rate (OR 0.85; 95% CI 0.50–1.45), nor the miscarriage rate (OR 1.24; 95% CI 0.71–2.19) showed statistically significant differences (Table 4 and Supplementary Figure S4). Sensitivity analyses aligned with these results (Table 4).

**Table 4:** Summary of results of meta-analyses of comparison between sequential transfer and blastocyst transfer.

Outcome indicator	Studies	Samples	OR (95% Cl)	*P* value	Heterogeneity	Adjusted OR (95% Cl)	Adjusted *p* value
Chemical pregnancy rate	2	322	1.32 (0.85–2.06)	0.22	0%	/	/
Clinical pregnancy rate	7	1,089	1.68 (1.12–2.53)	0.01	59%	1.80 (1.12–2.89)	0.02
Ongoing pregnancy rate	3	476	1.59 (0.61–4.13)	0.34	81%	1.50 (0.28–8.14)	0.64
Implantation rate	4	1,384	0.98 (0.67–1.41)	0.90	40%	1.01 (0.57–1.81)	0.96
Multiple pregnancy rate	7	402	0.85 (0.50–1.45)	0.56	19%	0.89 (0.45–1.74)	0.73
Miscarriage rate	6	355	1.24 (0.71–2.19)	0.45	0%	1.52 (0.78–2.97)	0.22

We conducted individualized subgroup analyses on patients diagnosed with RIF. Notably, the chemical pregnancy rate (OR 1.66; 95% CI 1.19–2.32) and the clinical pregnancy rate (OR 1.65; 95% CI 1.19–2.27) were significantly elevated in the sequential transfer group compared to the control group. Conversely, no significant differences emerged in ongoing pregnancy rate (OR 1.53; 95% CI 0.82–2.84), implantation rate (OR 1.44; 95% CI 0.90–2.29), multiple pregnancy rate (OR 1.08; 95% CI 0.72–1.61), or miscarriage rate (OR 1.40; 95% CI 0.80–2.44) between the groups (Table 5 and Supplementary Figure S5). Upon excluding the study conducted by Mengxia Ji et al., the ongoing pregnancy rate significantly improved in the sequential transfer group (OR 2.06; 95% CI 1.35–3.15). Similarly, when omitting the Koichi Kyono et al. study, the implantation rate also showed a significant increase (OR 1.70; 95% CI 1.07–2.70) in the sequential transfer group compared to the control group (Table 5). These findings suggest that caution should be exercised when interpreting these results until further evidence becomes available.

**Table 5:** Summary of results of meta-analyses of comparison in RIF patients.

Outcome indicator	Studies	Samples	OR (95% Cl)	*P* value	Heterogeneity	Adjusted OR (95% Cl)	Adjusted *p* value
Chemical pregnancy rate	4	600	1.66 (1.19–2.32)	0.003	0%	1.76 (1.08–2.88)	0.02
Clinical pregnancy rate	9	2099	1.65 (1.19–2.27)	0.002	56%	1.81 (1.31–2.49)	0.0003
Ongoing pregnancy rate	4	791	1.53 (0.82–2.84)	0.18	70%	2.06 (1.35–3.15)	0.0008
Implantation rate	5	4,104	1.44 (0.90–2.29)	0.13	82%	1.70 (1.07–2.70)	0.03
Multiple pregnancy rate	9	755	1.08 (0.72–1.61)	0.71	14%	1.04 (0.63–1.70)	0.88
Miscarriage rate	6	630	1.40 (0.80–2.44)	0.24	27%	1.32 (0.60–2.87)	0.49

## Discussion

### Principal findings

Sequential embryo transfers notably enhance pregnancy outcomes in infertile patients, with improvements most evident compared to cleavage embryo transfers. Furthermore, sequential embryo transfers have elevated both chemical and clinical pregnancy rates among patients diagnosed with RIF.

The evidence quality spanned from very low to high. This variance largely stems from the inherent nature of this review, which predominantly relies on observational studies. Additionally, the considerable heterogeneity observed across the studies can likely be attributed to diverse study populations.

### Study strengths

Our systematic review is comprehensive, encompassing 4,054 cases identified to date. Beyond merely comparing sequential embryo transfer to cleavage embryo transfer and blastocyst transfer, we also distinctly analyzed subgroups comprising RIF patients. This study adhered to the PRISMA guidelines, underscoring its methodological rigor. Additionally, we meticulously assessed the risk of bias within the included studies. Collectively, these factors considerably bolster the validity of our findings.

### Study limitations

Most of the studies incorporated into this review were observational. Significant variability was evident among the included studies, particularly in aspects such as study population, study duration, transfer protocol, patient BMI, cycle type, and the number of embryos transferred, as detailed in Table 1. Our results could have been influenced by the disparate transfer methodologies, the number of embryos transferred, and certain incomplete observations from some studies. A notable limitation was our inability to adjust for relevant confounders in our primary analyses, due to the absence of individual patient data—specifically metrics like obesity levels, hormone concentrations, and endometrial conditions. Furthermore, there was inconsistency in the definitions of observed outcome indicators across studies; notably, the definitions for RIF varied among the included studies.

### Interpretation of the results

Transferring embryos from the laboratory to the uterus is a pivotal phase in any ART cycle. Embryos were commonly transferred on day 3, during the cleavage stage; however, the last decade has seen a growing inclination toward day 5 blastocyst transfers (25). This shift to the blastocyst stage more closely mirrors the physiologically natural timing of implantation, fostering improved synchronization between embryonic development and the endometrial environment (25). It’s been documented that women who opt for blastocyst transfers exhibit higher clinical pregnancy and live birth outcomes compared to their counterparts who undergo cleavage stage embryo transfers (26). A caveat to this approach is that extended culture to the blastocyst stage might reduce the number of viable embryos suitable for transfer or cryopreservation, occasionally even leading to cycle cancelation.

Sequential embryo transfer offers a solution to this limitation. Preliminary data showcased a rise in pregnancy outcomes post sequential embryo transfers (4), although subsequent studies yielded mixed results (15). Our research showed that sequential embryo transfers bolstered the rates of chemical pregnancies, clinical pregnancies, and ongoing pregnancies in patients facing infertility. Our subgroup analysis revealed that sequential transfers led to superior chemical and clinical pregnancy rates when juxtaposed against cleavage stage transfers. Interestingly, this disparity diminished when pitted against blastocyst transfers, alluding to the potential benefits of blastocysts in elevating pregnancy outcomes. The heightened clinical pregnancy rates following sequential transfers, instead of mere blastocyst transfers, might be attributable to the nuanced two-step transfer protocol. The endometrium is optimally receptive to embryos within a concise timeframe, dubbed the “window of implantation” (27). It’s theorized that this “window” might oscillate during the luteal phase, thus pinpointing its precise dynamics is integral for embryo transfer scheduling (28). Sequential transfers aptly address this challenge by potentially enhancing the likelihood of embryos coinciding with this “implantation window” (29). Moreover, emerging evidence posits that the preliminary transferred cleavage-stage embryos can modulate the immune response and engage in pivotal interactions with immune cells within the uterus during sequential transfers. This paves the way for a more conducive endometrial setting for the subsequent transfer (30–32). It’s crucial to underscore that the exact definition of ongoing pregnancy rates might vary across studies. Given that ongoing pregnancy rates are intricately tied to various maternal factors—like basal metabolic conditions, hormonal levels, and endometrial health—the credibility of this metric warrants circumspection, especially in the absence of granular patient data. In addition, our study did not have significant data on live birth rates. Results on live birth rate are more valuable but were not reported in any of the included studies.

In our study, there was no significant difference in multiple pregnancy rates. Although logically, the multiple pregnancy rate should be higher for sequential embryo transfer, the multiple pregnancy rate is mainly related to the number of embryos transferred. Of all the included studies, excluding those in which the number of embryos transferred to patients was unclear, only one study showed that two embryos were transferred to patients in the sequential transfer group and one embryo was transferred to patients in the control group, whereas the rest of the studies had essentially the same number of embryos transferred to patients in the sequential transfer group and the control group. Therefore, it makes sense that there is no difference in multiple pregnancy rates.

Previous research has demonstrated that sequential embryo transfer considerably enhances pregnancy outcomes in patients with RIF (24, 33). Contrastingly, the same investigations revealed no marked improvement in clinical pregnancy rates for RIF patients undergoing sequential embryo transfer compared to day 5 blastocyst transfers (14). One plausible explanation for these divergent findings lies in the variations in the definitions of RIF and the inclusion criteria adopted across these studies. In our research, sequential embryo transfer yielded an uptick in both chemical and clinical pregnancy rates for RIF patients, even without distinguishing between cleavage-stage embryos and blastocysts.

## Conclusion

Our systematic review and meta-analysis confirm that sequential transfer may enhance clinical pregnancy rate in a small subgroup of well-selected women. Women with an adequate number of embryos will do equally good with blastocyst transfer. Guidance should be developed for clinicians to decide who should be considered for sequential transfer based on grade and number of available embryos and clinical history. Nonetheless, precise definitions for metrics such as the chemical pregnancy rate, ongoing pregnancy rate, and implantation rate need to be standardized, especially when considering patients with RIF. Furthermore, comprehensive data collection, encompassing a broader spectrum of patient information, is pivotal for more in-depth and rigorous analyses in future research.

## Data availability statement

The original contributions presented in the study are included in the article/Supplementary material, further inquiries can be directed to the corresponding author/s.

## Author contributions

WT: Writing – original draft, Data curation, Methodology, Software. HX: Writing – original draft, Software. FW: Writing – review & editing, Data curation. YW: Methodology, Validation, Writing–review–&–editing. XM: Data curation, Writing – review & editing. XZ: Conceptualization, Writing – review & editing. XS: Resources, Writing – review & editing. JY: Supervision, Validation, Writing – review & editing.

## Figures and Tables

**Table:** 

ART	Assisted reproductive technique
BMI	Body mass index
CCT	Case–control trial
ET	Embryo transfer
FET	Frozen embryo transfer
GRADE	Grading of recommendations assessment, development and evaluation
ICSI	Intracytoplasmic sperm injection
IVF-ET	*In vitro* fertilization and embryo transfer
OR	Odds ratio
PC	Prospective cohort
PRISMA	Preferred reporting items for systematic reviews and meta-analyses
RC	Retrospective cohort
RCT	Randomized controlled trial
RIF	Repeated implantation failure
