# Supplementary material for: Effect of sequential embryo transfer on in vitro fertilization and embryo transfer outcomes: a systematic review and meta-analysis
Source: Front Med (Lausanne). 2023 Dec 19;10:1303493. doi: 10.3389/fmed.2023.1303493 (PMC10758412; doi:10.3389/fmed.2023.1303493)
Supplement: Supplementary file 1 [file Data_Sheet_1.docx]

**Supplementary Figure**

**
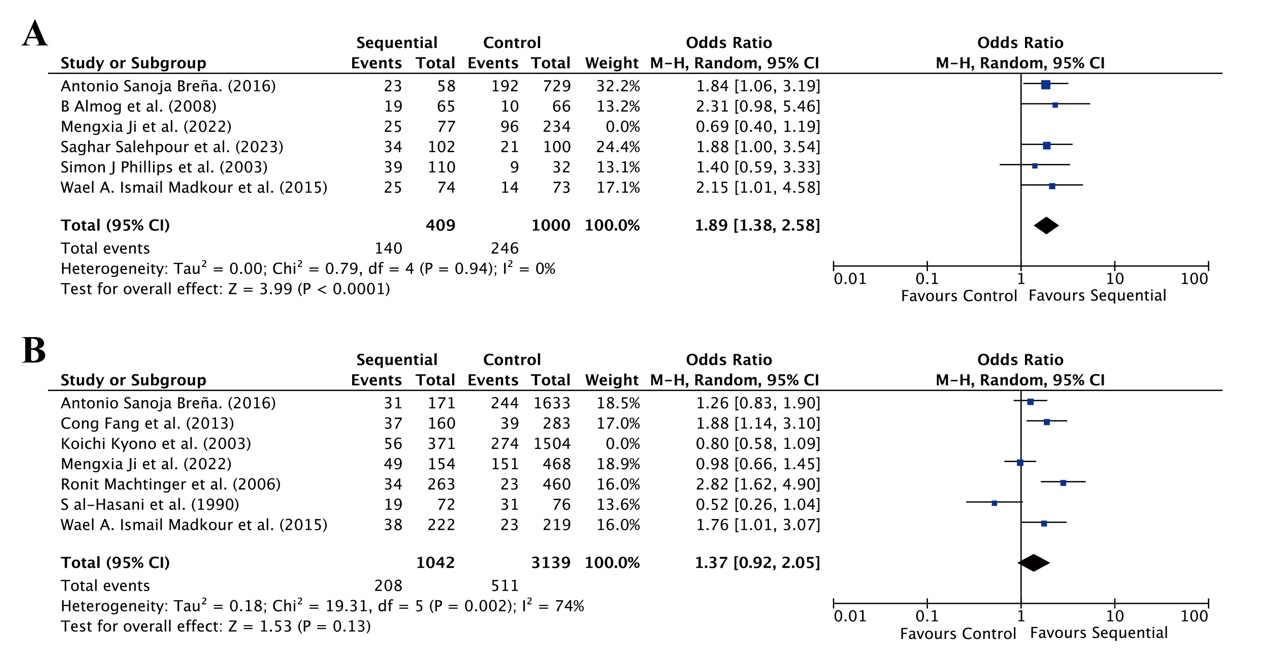
**

**Figure S1. Adjusted ORs for sequential transfer group and control group.** A. Ongoing pregnancy rate, B. Implantation rate.

**
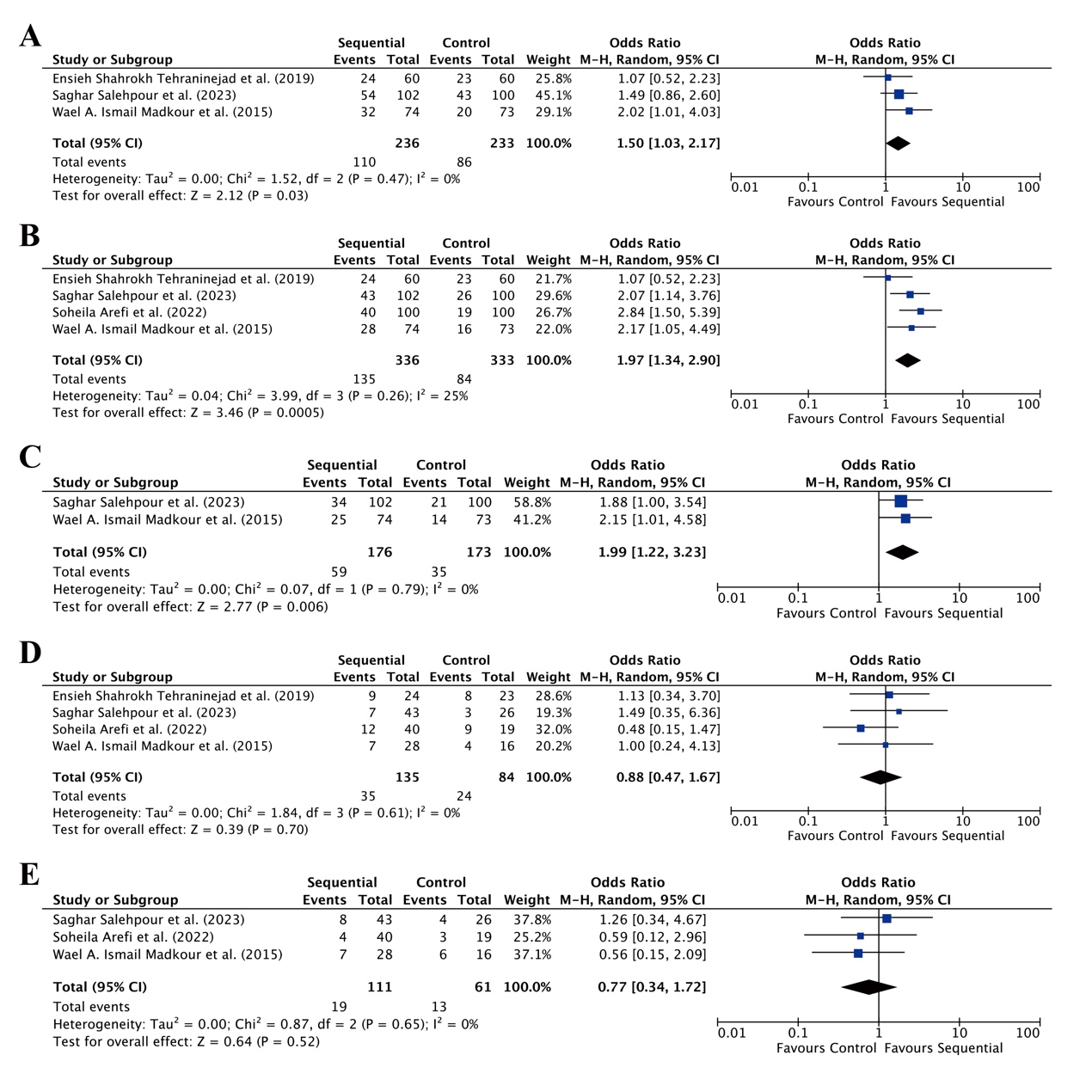
**

**Figure S2. RCT subgroup analysis.** A. Chemical pregnancy rate, B. Clinical pregnancy rate, C. Ongoing pregnancy rate, D. Multiple pregnancy rate, E. Miscarriage rate.


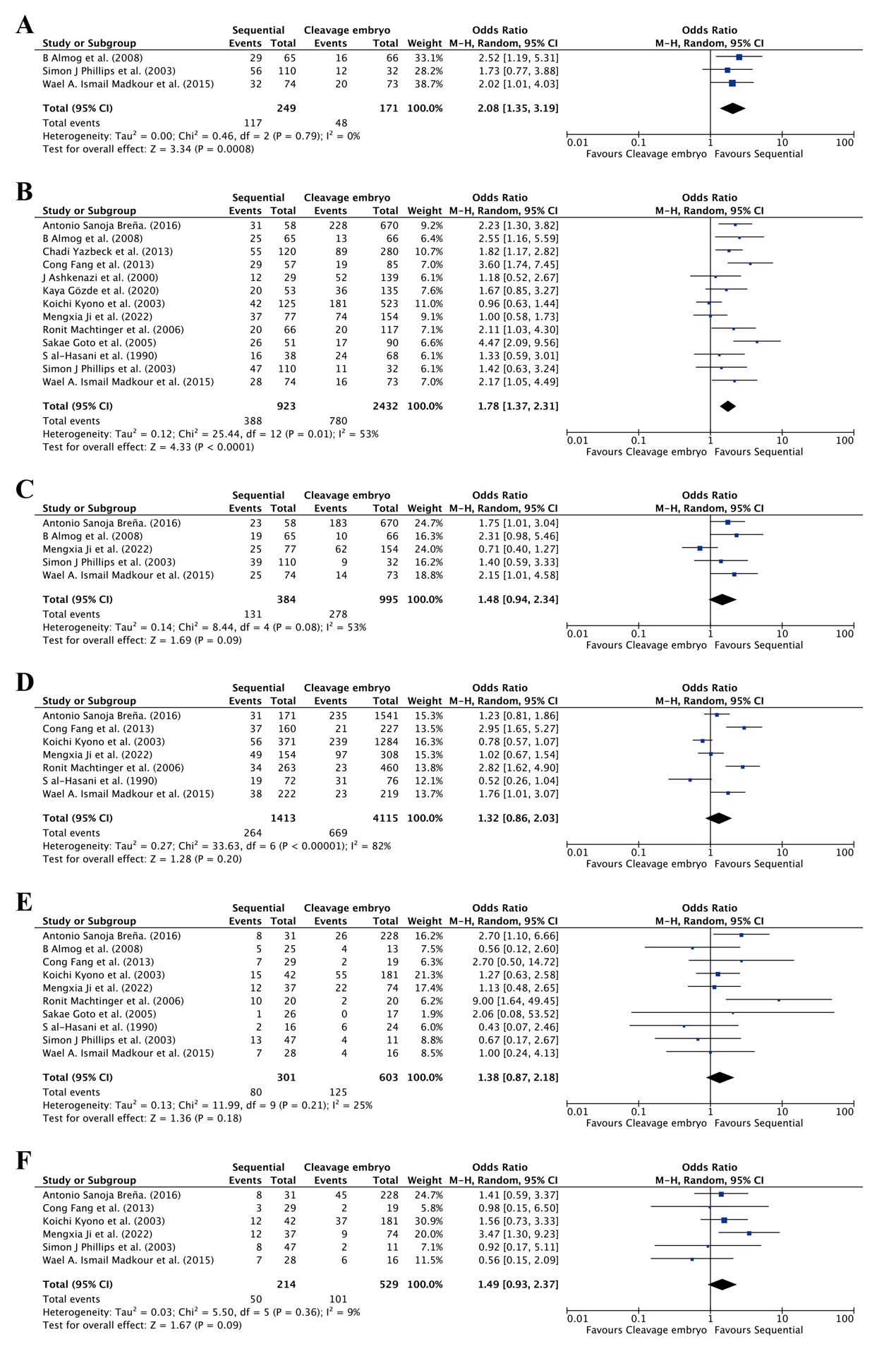


**Figure S3. Differences in assisted reproductive outcomes between sequential transfer group and cleavage embryo group.** A. Chemical pregnancy rate, B. Clinical pregnancy rate, C. Ongoing pregnancy rate, D. Implantation rate, E. Multiple pregnancy rate, F. Miscarriage rate.


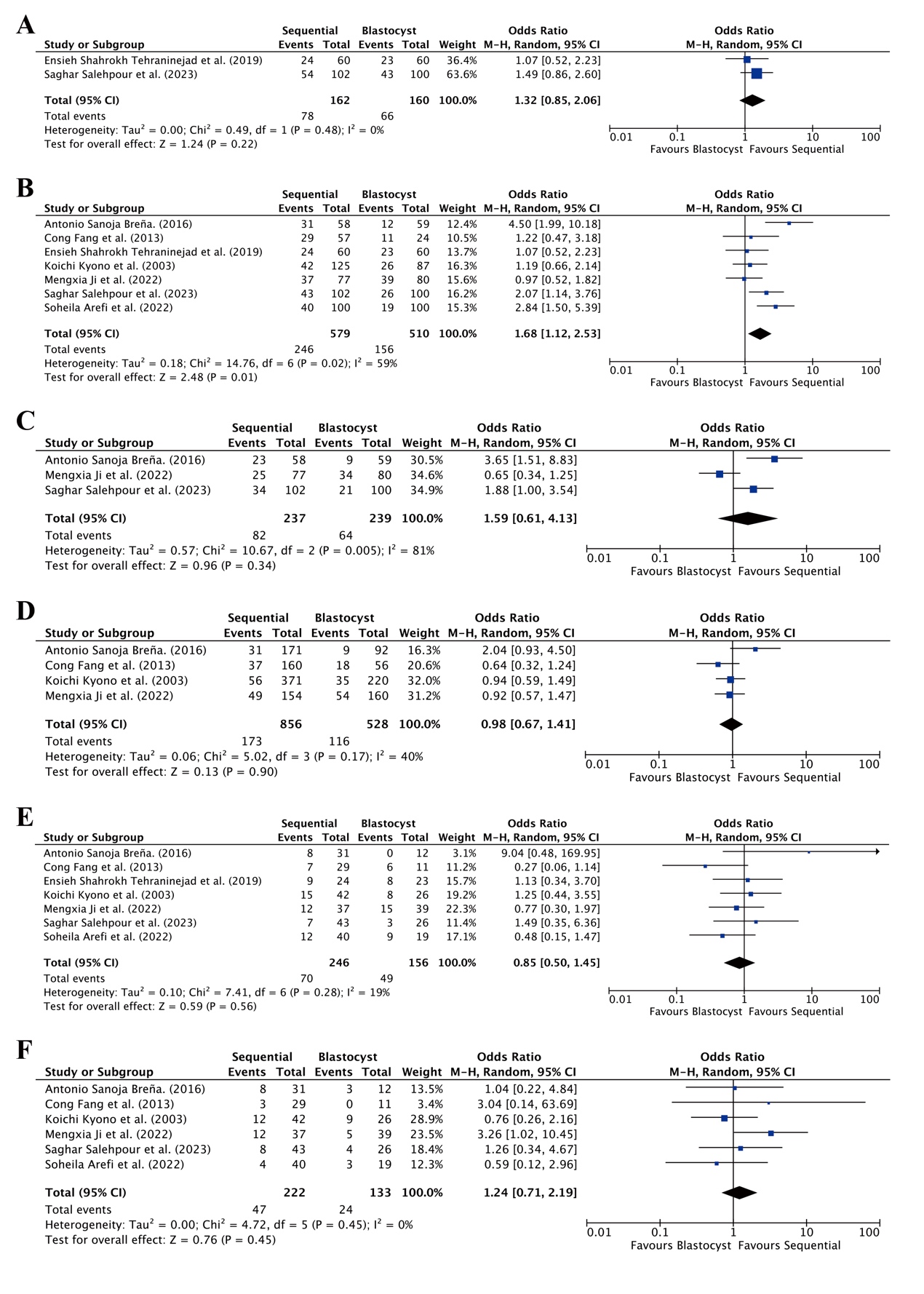


**Figure S4. Differences in assisted reproductive outcomes between sequential transfer group and blastocyst group.** A. Chemical pregnancy rate, B. Clinical pregnancy rate, C. Ongoing pregnancy rate, D. Implantation rate, E. Multiple pregnancy rate, F. Miscarriage rate.


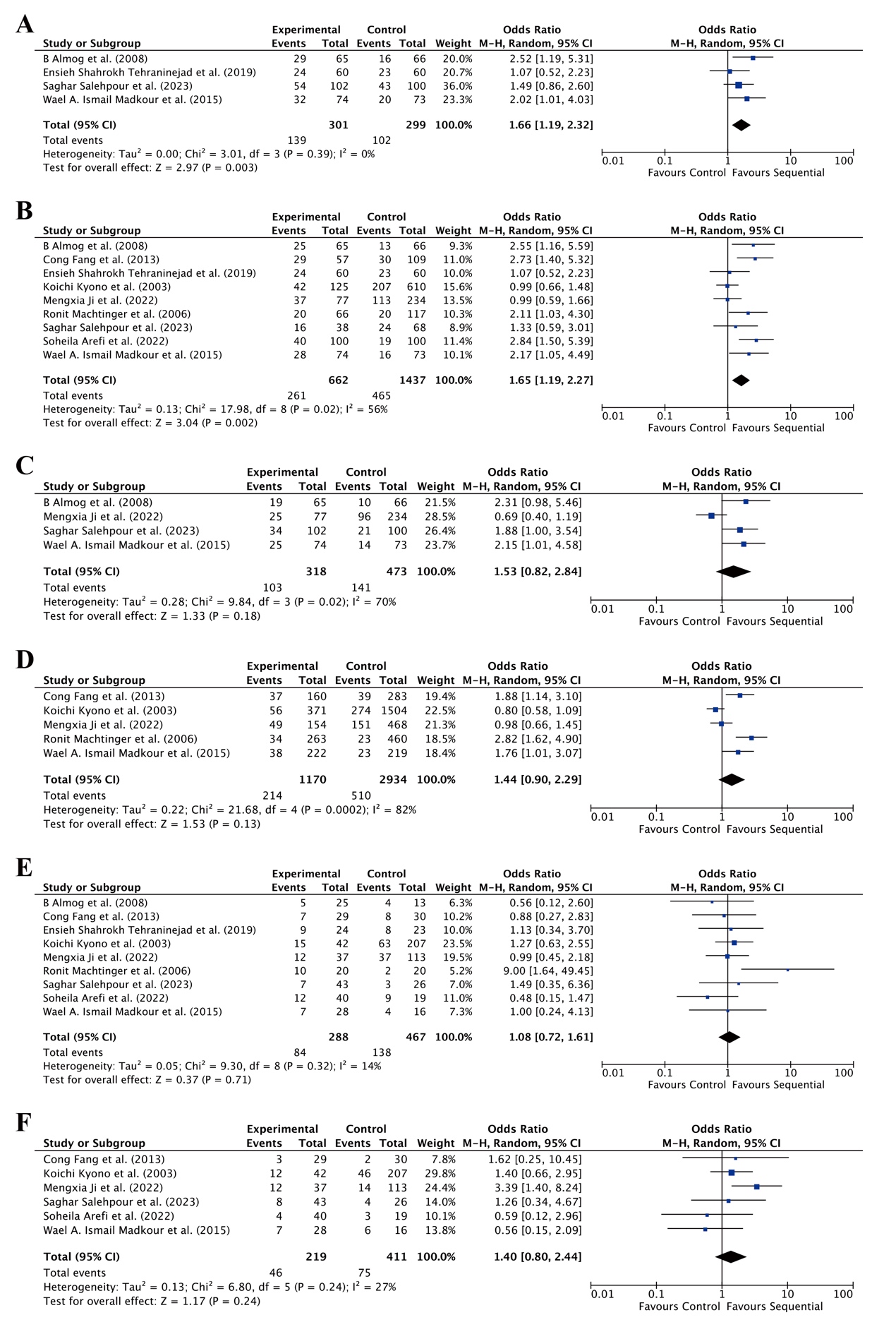


**Figure S5. Differences in assisted reproductive outcomes in RIF patients.** A. Chemical pregnancy rate, B. Clinical pregnancy rate, C. Ongoing pregnancy rate, D. Implantation rate, E. Multiple pregnancy rate, F. Miscarriage rate.
