# Supplementary material for: Effect of sequential embryo transfer on in vitro fertilization and embryo transfer outcomes: a systematic review and meta-analysis
Source: Front Med (Lausanne). 2023 Dec 19;10:1303493. doi: 10.3389/fmed.2023.1303493 (PMC10758412; doi:10.3389/fmed.2023.1303493)
Supplement: Supplementary file 2 [file Table_1.docx]

**Supplementary Table**

**Table S1. Quality and risk of bias assessment.**

| Study | Random sequence generation (selection bias) | Allocation concealment (selection bias) | Blinding of participants and personnel (performance bias) | Blinding of outcome assessment (detection bias) | Incomplete outcome data (attrition bias) | Selective reporting (reporting bias) | GRADE |
| --- | --- | --- | --- | --- | --- | --- | --- |
| Antonio Sanoja Breña. (2016) | High risk of bias | High risk of bias | High risk of bias | High risk of bias | Unclear risk of bias | Unclear risk of bias | Very low |
| B Almog et al. (2008) | Unclear risk of bias | High risk of bias | High risk of bias | High risk of bias | High risk of bias | Unclear risk of bias | Very low |
| Chadi Yazbeck et al. (2013) | High risk of bias | High risk of bias | High risk of bias | High risk of bias | High risk of bias | Unclear risk of bias | Very low |
| Cong Fang et al. (2013) | Unclear risk of bias | High risk of bias | High risk of bias | High risk of bias | High risk of bias | Unclear risk of bias | Very low |
| Ensieh Shahrokh Tehraninejad et al. (2019) | Low risk of bias | Unclear risk of bias | High risk of bias | Unclear risk of bias | High risk of bias | Unclear risk of bias | Low |
| J Ashkenazi et al. (2000) | High risk of bias | High risk of bias | High risk of bias | High risk of bias | High risk of bias | Unclear risk of bias | Very low |
| Kaya Gözde et al. (2020) | Unclear risk of bias | High risk of bias | High risk of bias | High risk of bias | High risk of bias | Unclear risk of bias | Very low |
| Koichi Kyono et al. (2003) | High risk of bias | High risk of bias | High risk of bias | High risk of bias | High risk of bias | Unclear risk of bias | Very low |
| Mengxia Ji et al. (2022) | Low risk of bias | Unclear risk of bias | Unclear risk of bias | Unclear risk of bias | High risk of bias | Unclear risk of bias | Moderate |
| Ronit Machtinger et al. (2006) | High risk of bias | High risk of bias | High risk of bias | High risk of bias | High risk of bias | Unclear risk of bias | Very low |
| S al-Hasani et al. (1990) | High risk of bias | High risk of bias | High risk of bias | High risk of bias | High risk of bias | Unclear risk of bias | Very low |
| Saghar Salehpour et al. (2023) | Low risk of bias | Unclear risk of bias | Low risk of bias | Low risk of bias | Low risk of bias | Unclear risk of bias | High |
| Sakae Goto et al. (2005) | High risk of bias | High risk of bias | High risk of bias | High risk of bias | High risk of bias | Unclear risk of bias | Very low |
| Simon J Phillips et al. (2003) | High risk of bias | High risk of bias | High risk of bias | High risk of bias | Unclear risk of bias | Unclear risk of bias | Very low |
| Soheila Arefi et al. (2022) | Low risk of bias | Unclear risk of bias | Unclear risk of bias | Unclear risk of bias | High risk of bias | Unclear risk of bias | Moderate |
| Wael A. Ismail Madkour et al. (2015) | Low risk of bias | Unclear risk of bias | Low risk of bias | Low risk of bias | High risk of bias | Unclear risk of bias | Moderate |
